# The complete mitochondrial genome of *Fopius arisanus* (Sonan 1932) (Hymenoptera: Braconidae)

**DOI:** 10.1080/23802359.2021.2009383

**Published:** 2021-12-17

**Authors:** Pumo Cai, Deqing Yang, Xuxing Hao, Guoqing Yue, Lili Jiang, Kang Xiao, Pingfan Jia, Jianquan Yang, Qing’e Ji, Jia Lin

**Affiliations:** a Department of Horticulture, College of Tea and Food Science, Wuyi University, Wuyishan, China; b Institute of Biological Control, Plant Protection College, Fujian Agriculture and Forestry University, Fuzhou, China; c Key Laboratory of Biopesticide and Chemical Biology, Ministry of Education, Fuzhou, China; d State Key Laboratory of Ecological Pest Control for Fujian and Taiwan Crops, Fuzhou, China

**Keywords:** *Fopius arisanus*, tephritid, mitochondrial genome, phylogenetic tree

## Abstract

*Fopius arisanus* (Sonan, 1932), an important egg parasitoid of several notorious tephritid pests, plays a key role in biological control programs. In the present study, the whole mitochondrial genome of *F*. *arisanus* was sequenced and characterized. The mitogenome of *F*. *arisanus* is 16,425 bp in length with 14.94% GC content, and contains 13 protein-coding genes (PCGs), 22 transfer RNA genes (tRNAs), and two ribosomal RNA genes (rRNAs). The phylogenetic trees demonstrated that *F*. *arisanus* is sister group to *Psyttalia concolor, P. humilis, P. lounsburyi* and *Diachasmimorpha longicaudata.*

*Fopius arisanus* (Sonan, 1932) (Hymenoptera: Braconidae) parasitizes more than 40 species of tephritid pest (Cai et al. 2017). Owing to its unique characteristics for parasitizing eggs, *F. arisanus* has become a key component of biological control programs that aim to suppress tephtirid pest populations and therefore reduce economic loss (Vargas et al. 2001). However, to date, there are few studies detailing its genome information. Hence, in this study, we determined the complete mitochondrial genome of *F. arisanus* and analyzed the evolutionary relationship between *F. arisanus* and other braconid wasps.

The samples were obtained from Fujian Agriculture and Forestry University (26.084220°N, 119.231164°E), Fuzhou City, Fujian Province, China. The voucher specimens (20190808FA) were deposited at the Fujian Agriculture and Forestry University (URL: http://zbxy.fafu.edu.cn; contact: Qinge Ji, jiqinge@fafu.edu.cn). Total DNA was extracted using CTAB extraction method (Vanzyme, Nanjing, China) and a 400-bp insert library was constructed. An Illumina Novaseq 6000 platform in 150 bp paired-end read mode was used to sequence the constructed library. Filtering of raw data was performed in fastp v.0.20.0 (Chen et al. 2018) resulting in 21,648,152 clean reads, which were then assembled by SPAdes v.3.9.0 software (Bankevich et al. 2012). Annotation of the assembled sequence was performed using MITOS web server (Bernt et al. 2013).

The complete mitochondrial genome of *F. arisanus* is 16,425 bp in length, containing 13 protein-coding genes (PCGs), two ribosomal RNA genes (rRNAs), 22 transfer RNA genes (tRNAs), and a non-coding region (control region). The mitogenome comprises 39.37% A, 9.14% G, 5.8% C, and 45.68% T, with a significant A + T (85.06%) bias. For the PCGs, six genes (*cox*2, *atp*8, *nad*2, *nad*4*l*, *nad*5, *nad*6) had a start codon of ATT, four (*cox*1, *cox*3, *atp*6, *cob*) had ATG, and three had ATA (*nad*1, *nad*3, *nad*4). All PCGs contained the stop codon TAA, except for *nad*3 which had TAG.

We analyzed the nucleotide sequences of PCGs using the Maximum-Likelihood (ML) and Bayesian Inference (BI) approaches to understand the phylogenetic relationship of *F. arisanus* with 10 other species and to estimate the phylogenetic position of *F. arisanus*. Two species respectively from Sigalphinae and Pselaphaninae were used as outgroups (Lyu et al. 2020). Phylogenetic analyses were performed with Bayesian inference in MrBayes 3.2.3 (Ronquist et al. 2012) and maximum likelihood in RAxML 8.2.10 (Stamatakis 2014). The phylogenetic trees showed that *F. arisanus* clustered with *Psyttalia concolor, P. humilis, P. lounsburyi* and *Diachasmimorpha longicaudata* as a separated clade. To date, studies that relate to the genome analysis of *F. arisanus* are limited, and therefore we hope that our data provides valuable information for further studies (Figure 1).

**Figure 1.**
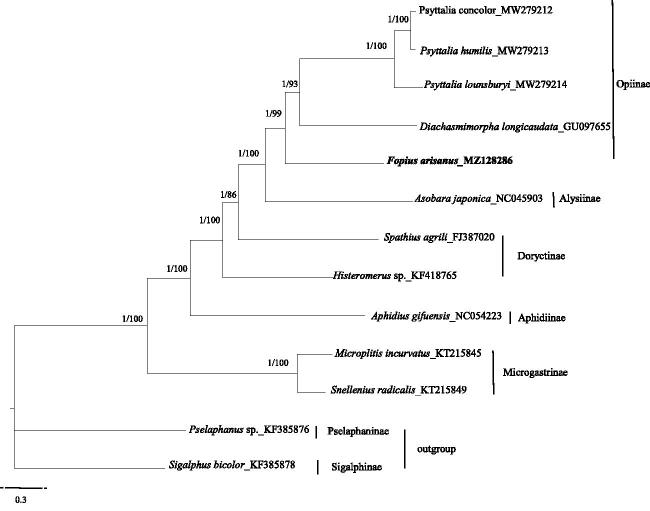
Phylogenetic relationships among subfamilies of the Braconidae inferred from nucleotides of 13 PCGs using Bayesian and maximum-likelihood (ML) methods (GenBank accession numbers provided). The Bayesian posterior probabilities (PP) and bootstrap support (BS) are marked besides the nodes.

## Data Availability

The complete mitochondrial genome sequence of *F. arisanus* is deposited in the GenBank database under the accession number MZ128286. The associated BioProject, SRA, and Bio-Sample numbers are PRJNA734964, SRS9131624, and SAMN19551218, respectively The Web link is https://www.ncbi.nlm.nih.gov/
